# Analysis of the association between microbiota and flavor formation during Zizhong Dongjian fermentation process

**DOI:** 10.1002/fsn3.4460

**Published:** 2024-10-17

**Authors:** Zhang Li, Miao Wang, Zhirong Yang

**Affiliations:** ^1^ Laboratory of Molecular Oncology, Frontiers Science Center for Disease‐related Molecular Network, State Key Laboratory of Biotherapy and Cancer Center, West China Hospital Sichuan University Chengdu China; ^2^ Laboratory Animal Center, West China School of Basic Medical Science & Forensic Medicine Sichuan University Chengdu China; ^3^ Key Laboratory of Biological Resource and Ecological Environment of the Ministry of Education, College of Life Sciences Sichuan University Chengdu China

**Keywords:** fermented vegetable, microbial community, network analysis, volatile flavor compounds, Zizhong Dongjian

## Abstract

Zizhong Dongjian (ZZDJ) is one of the most famous and popular fermented vegetables in China. The aim of this study was to explore the microbial communities and volatile flavor compounds of ZZDJ during different fermentation periods, as well as to reveal the potential correlation between microbiota and flavor. A total of 84 volatile flavor compounds were detected in 0‐year to 3‐year ZZDJ samples. Hydrocarbons were the most abundant flavor compounds in 0‐year and 1‐year samples, while esters became the predominant flavor components in 2‐year and 3‐year samples. Furthermore, *Loigolactobacillus*, *Pseudomonas*, and *Virgibacillus* were most predominant bacteria during the fermentation process of ZZDJ. Interestingly, all the fungi identified were yeasts. Among them, *Zygosaccharomyces* and *Symmetrospora* dominated alternatively throughout the fermentation process of ZZDJ. Through analysis of relativity between flavor compounds and microorganism of ZZDJ, we found that *Uncultured Pseudomonas* sp., *Virgibacillus sediminis*, *Zygosaccharomyces rouxii*, and *Symmetrospora marina* might play important roles in flavor information of ZZDJ.

## Graphical abstract

Dominant bacteria were found during Zizhong Dongjian (ZZDJ) fermentation process. Dynamic fungal communities were reported first during the ZZDJ fermentation process. A total of 84 volatile flavor compounds were detected during the ZZDJ fermentation process. This is the first report about the correlation between flavor and microorganisms of ZZDJ.

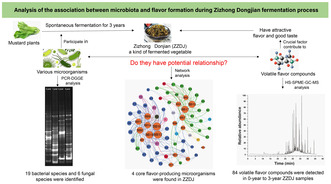

## 1 INTRODUCTION

Zizhong Dongjian (ZZDJ) is a traditional pickled vegetable from Zhizhong County, Sichuan Province of China. It is popular because of its attractive flavor and good taste (Zhang et al., 2022). ZZDJ is produced by spontaneous fermentation, which lasts for 2–3 years (Yao et al., 2015; Zhang et al., 2022). This spontaneous fermentation process relies highly on various microorganisms deriving from mustards’ surfaces and processing environments. Microorganisms play important roles in the quality and features of fermented food. Therefore, more and more attention is paid to the microbial communities in fermented vegetables (Lee et al., 2005; Wu et al., 2015; Xiong et al., 2012). Previous reports indicate that the dominant bacteria in various fermented vegetables belonged to the phyla *Proteobacteria* and *Firmicutes* (An et al., 2021; Liang et al., 2018; Liu, Li, Huang, et al., 2019; Zhang et al., 2022). Among bacteria, lactic acid bacteria (LABs) existed widely in fermented vegetables, such as kimchi, Pao cai, Suan‐cai, and Jiang‐shui (Lee et al., 2017; Liang et al., 2018; Liu, Li, Wei, et al., 2019). To our knowledge, the microbial communities during ZZDJ fermentation are not fully elucidated yet.

The volatile flavor compounds are important to the aroma and flavor of fermented vegetables (Sonmezdag et al., 2019). The production of volatile flavor compounds is affected by various factors, such as raw materials, fermentation methods, and microorganisms. Among them, microbiota contributes the most to the flavor formation of fermented vegetables (Liang, He, Wang, Song, Chen, Lin, Ji, & Zhang, 2020b). Nowadays, more and more attention is paid to the correlation between microbial communities of fermented vegetables and their flavor (Liu, She, et al., 2020; Zhang, Chen, et al., 2019; Zhang, Zhang, et al., 2021). For instance, *Lactobacillus* is associated with the production of dominant esters and terpenes in several fermented vegetables (Xiao et al., 2020). *Zygosaccharomyces rouxii* is used widely as a starter in fermented food production, which could promote flavor information of fermented food (Devanthi et al., 2018; Lee et al., 2013; Liu, Wang, et al., 2020). Like most traditionally fermented vegetables, ZZDJ is still made based on worker's experience under non‐sterile and open environments, which make it difficult to maintain stable flavor and quality between different batches. There is little information available concerning the connection between microbiota and flavor during the fermentation process of ZZDJ. Thus, it is important to elucidate the microbial communities and flavor compounds of ZZDJ and their potential relationship.

In the present study, the analysis of microbiota and volatiles of ZZDJ was performed using polymerase chain reaction–denaturing gradient gel electrophoresis (PCR–DGGE) and solid‐phase‐gas chromatography–mass spectrometry (SPME‐GC–MS). Our aim is to explore the microbial communities and volatile flavor compounds of ZZDJ during its different fermentation periods and reveal the potential relationship between them. Our study might provide theoretical foundations for improving the quality and flavor of ZZDJ.

## 2 MATERIALS AND METHODS

### 2.1 Sample collection

All the samples of ZZDJ were collected from a well‐known local enterprise located in Zizhong county, Sichuan province of China. All the raw materials came from the same batch. Samples (0, 1, 2, and 3 years) were collected from sealed jars at 1‐year interval during the ZZDJ fermentation process. Samples were then sealed in aseptic plastic bags and stored at −80°C for further analysis.

### 2.2 DNA extraction from ZZDJ samples

Total DNA of bacteria and fungi was extracted from ZZDJ samples using a rapid DNA extraction kit (BioTeke Corporation, Beijing, China) according to the manufacturer's instructions. The concentration and purity of DNA were assayed by NanoDrop 2000 (Thermo Fisher Scientific, Wilmington, MA, USA). The integrity was checked using 1% agarose gel electrophoresis. Then, the extracted DNA was stored at −80°C for further analysis.

### 2.3 Microbial community analysis through PCR–denaturing gradient gel electrophoresis (DGGE)

In order to obtain suitable DNA fragments for DGGE analysis, nested PCR was carried out to amplify target genes in two steps. First, the bacterial 16S rRNA gene was amplified with primers 27F and 1492R. The fungal 26S ribosomal RNA (rRNA) gene was amplified with primers NL1F and NL4R. In the second step, the 16S ribosomal DNA (rDNA) V3 region was amplified with primers GC357F and 517R, and the fungal 26S rRNA D1 region was amplified with primers GCNL1F and LS2R (Chang et al., 2008; Haruta et al., 2006). Primers used in DGGE are listed in Table 1. The PCR procedures were given as follows: initial denaturation at 94°C for 4 min; 30 cycles of denaturation at 94°C for 30 s, annealing at 56°C for 1 min, and extension at 72°C for 30 s; and final extension at 72°C for 7 min.

**TABLE 1:** Primers used in this study.

Sequence no.	Name of primer	Primer sequence
1	27F	AGAGTTTGATCCTGGCTCAG
2	1492R	CGGCTACCTTGTTACGACTT
3	NL1F	GCATATCAATAAGCGGAGGAAAAG
4	NL4R	GGTCCGTGTTTCAAGACGG
5	GC357F	CGCCCGCCGCGCGCGGCGGGCGGGGCGGGGG
		CACGGGGGGCTACGGGAGGCAGCAG
6	517R	ATTACCGCGGCTGCTGG
7	GCNL1F	CGCCCGCCGCGCGGCGGGCGGGGCGGGGGCG
		CATATCAATAAGCGGAGGAAAAG
8	LS2R	ATTCCCAAACAACTCGACTC

The PCR products were separated by denaturing gradient gel electrophoresis (DGGE) using a DCode Universal Mutation Detection System (Bio‐Rad, Richmond, CA, USA). Briefly, 35 μL PCR products and 5 μL loading buffer were mixed and added to 8% (w/v) polyacrylamide gel. Optimal separation was achieved with a 45%–65% urea–formamide denaturing gradient for both bacterial and fungal communities (100% denaturant is identified as 40% (v/v) formamide and 7 M urea). The electrophoresis was performed at 80 V for 14 h at 60°C. Then, the gels were stained with 1*SYBR Green I (Molecular Probes, Eugene, UK) for 30 min and viewed under ultraviolet (UV) light. Bands with the same migration position in different lanes were generally considered to be the same microbial species (Lv et al., 2012). Major bands were excised from the gels and eluted overnight in 50 μL ultrapure water at 4°C. Subsequently, the eluted DNA was re‐amplified with the same primers without the GC clamp. The purified amplicons were sent to a biotech company (Shanghai Sangon Biological Engineering Technology & Services Co., Ltd) for sequencing. Sequences were blasted in GenBank database to identify the closest related species.

### 2.4 Volatile profiles analyzed by headspace solid‐phase microextraction (HS‐SPME) coupled with gas chromatography–mass spectrometry (GC–MS)

Volatile flavor compounds in ZZDJ samples were extracted by HS‐SPME (Supelco Inc., Bellefonte, PA, USA) and analyzed by GC–MS (Agilent Technologies, Santa Clara, CA, USA). The HS‐SPME sampling condition was used as follows: Briefly, 10 g ZZDJ samples were cut into small pieces and added into a 20 mL headspace vial. The vial was tightly capped, and then, the sample vial was placed into a water bath at 65°C. In each extraction, the sample was kept for 30 min in disposal to achieve the partition equilibration. After this time, a 1 cm–50/30 μm divinyl‐benzene/carboxen/polydimethylsiloxane fiber (DVB/CAR/PDMS) (Supelco Inc., Bellefonte, PA, USA) was inserted into the vial septum and exposed to head space 1 cm above the sample to absorb the analytes. After 40 min, the fiber was withdrawn into the needle (Huang, Guo, et al., 2019; Selli et al., 2012; Zhao et al., 2021). For desorbing the adsorbates, the fiber was inserted into the GC injector at 250°C for 5 min and the injection mode was splitless.

Volatile flavor compounds were analyzed using an Agilent GC 6890N coupled to an Agilent MS 5975 and equipped with a DB‐wax capillary column (30.0 m × 0.25 mm × 0.25 μm, Agilent Technologies, Santa Clara, CA, USA). GC–MS conditions were as follows: the helium was used as a carrier gas at a flow rate of 1 mL/min. The initial temperature was held at 40°C for 5 min, and it was increased at a rate of 5°C/min to 220°C, which was kept for 5 min. The mass spectrum (MS) was operated in the electron impact mode at 70 eV ionization energy, and the temperature of ion source was 230°C. The scanning range of mass spectrum was 35–400 amu (atomic mass unit). The volatile compounds were identified by comparing mass spectrum (MS) and retention index (RI) with the data in the National Institute of Standards and Technology (NIST) database (NIST 14.0). RI was measured using C7–C40 alkane standards (Sigma‐Aldrich, Steinheim, Germany) under the same conditions. The relative intensity of each compound was calculated as the ratio between the area of the specific molecule and the sum of the areas of all identified peaks (peak area normalization method) in the chromatogram (Selli et al., 2012).

### 2.5 Statistical analysis

Denaturing gradient gel electrophoresis (DGGE) statistical analysis was performed using Quantity One software (vers. 4.0.1, Bio‐Rad, USA) according to the operations manual. The ratio of intensity in each band to all the bands in the same lane was identified as the relative intensity of each band. The Shannon diversity index (H) and the evenness index (E) were calculated based on the relative intensity and the number of bands. Based on the neighbor‐joining method, the phylogenetic tree was generated by Mega‐11 software (http://www.megasoftware.net/) with 1000 bootstrap replicates. To analyze microbial and volatile profiles, principal component analysis (PCA) was conducted using SIMCA‐14.1 software (Umetrics, Sweden). The distributions of microbiota and volatile compounds were visualized using heatmap generated through TBtools (Chen et al., 2020). To explore the relationship between microbiota and volatile compounds, and the relationship between bacteria and fungi, Spearman's correlation coefficient (*r*) was calculated using SPSS Statistics‐19.0 (SPSS Inc., USA). |*r*| >0.6 with statistical significance (*p* < .05) was considered as strong correlation (Zhu et al., 2018). The correlation networks were visualized using Gephi software (v 0.9.2).

## 3 RESULTS

### 3.1 Analysis of volatile flavor compounds during the ZZDJ fermentation process

**FIGURE 1:**
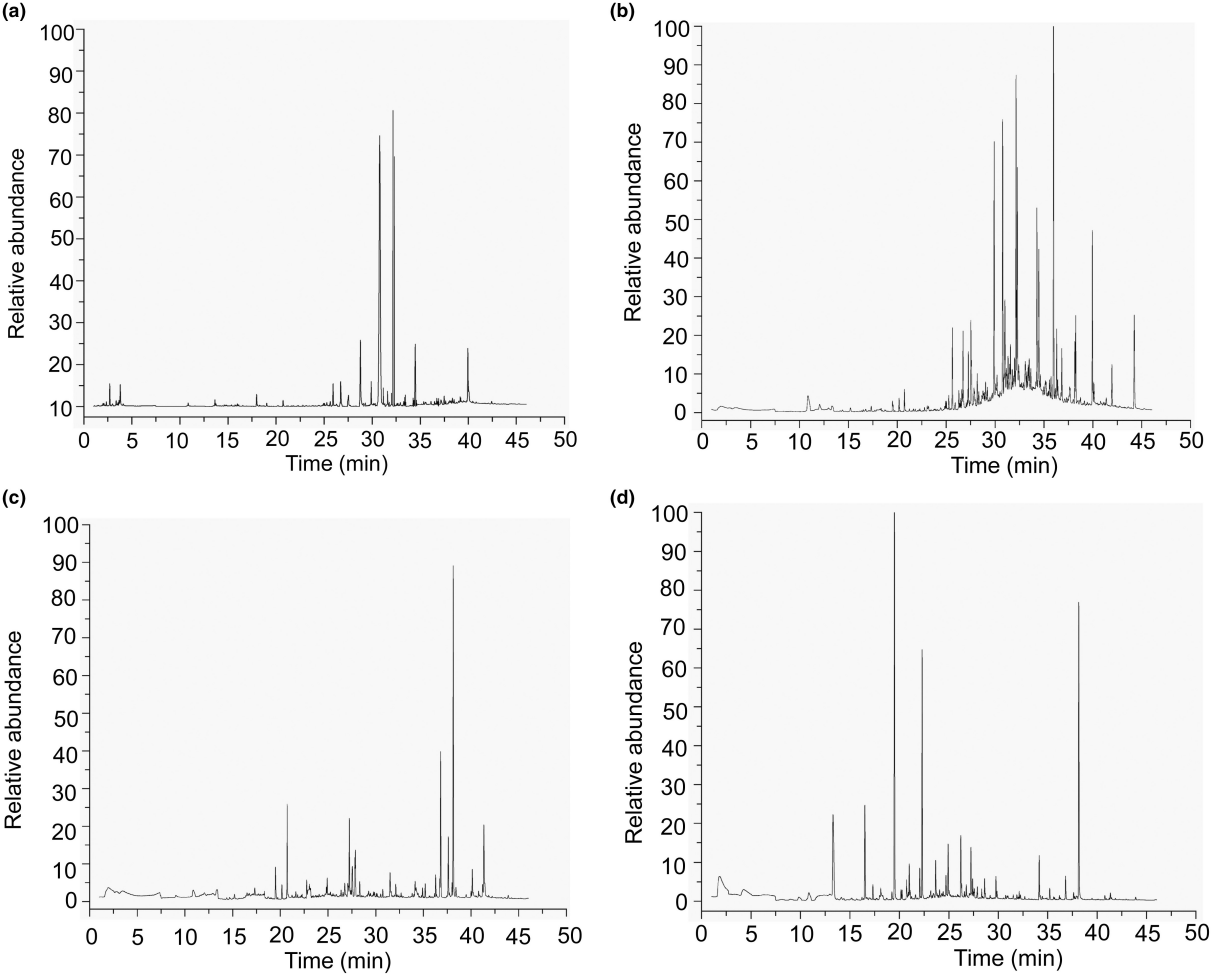
Chromatogram profiles of volatile compounds extracted using HS‐SPME‐GC–MS from the samples of 0–3 year ZZDJ.

**FIGURE 2:**
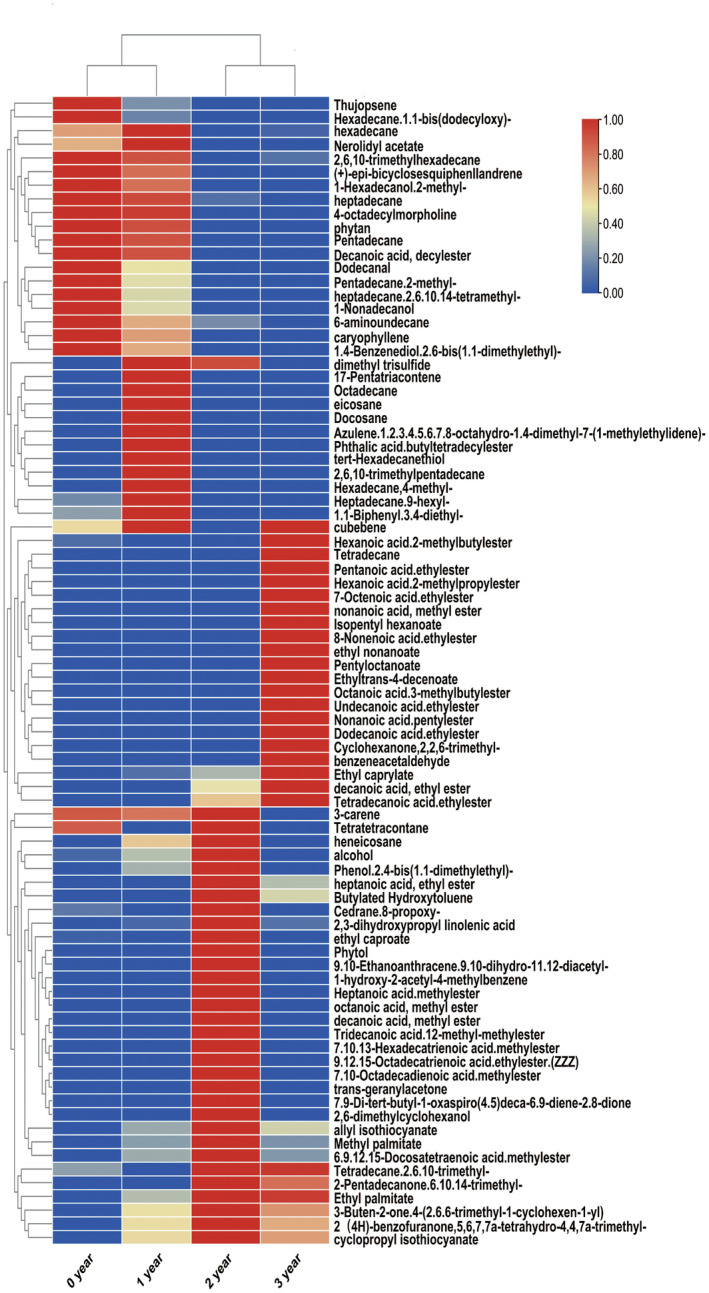
Heatmap plot of the volatile flavor compounds present in the four ZZDJ samples from different fermentation periods of ZZDJ. The color intensity is proportional to the relative abundance of volatile compounds.

**FIGURE 3:**
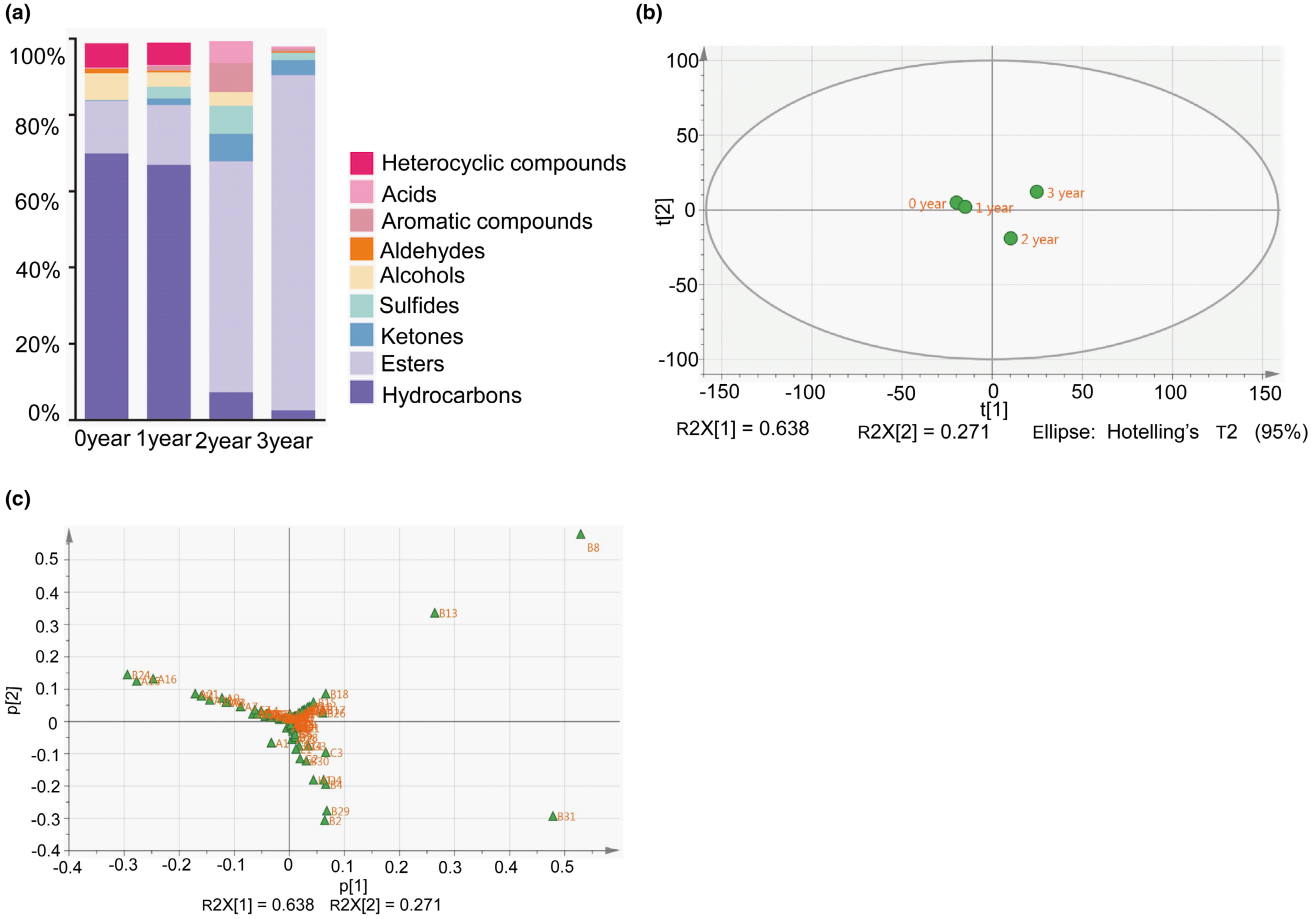
(a) The content of nine types of volatile compounds present in ZZDJ. Score (b) and loading (c) plots of the two components (PC1 vs. PC2) after principal component analysis (PCA) based on the relative abundance of the volatile compounds in 0–3 year ZZDJ samples. The different types of volatile compounds are designated by the letters A–I, and the abbreviation of each compound is given in Table S1.

Volatile profiles during the fermentation process of ZZDJ were analyzed by HS‐SPME‐GC–MS (Figure 1). The dynamic variation and evolution of volatile flavor compounds during ZZDJ fermentation are shown in the heatmap (Figure 2). A total of 84 volatile compounds were detected and classified into nine categories, namely, hydrocarbons, esters, ketones, sulfides, alcohols, aldehydes, aromatic compounds, acids, and heterocyclic compounds (Table S1). Specifically, there were 34, 45, 39, and 36 kinds of identified compounds in 0‐, 1‐, 2‐, and 3‐year ZZDJ samples, respectively. As shown in Figure 3a, the types and relative contents of volatile substances varied dramatically during the ZZDJ fermentation process. Hydrocarbons were the most abundant compounds in 0‐year and 1‐year samples, which accounted for 69.94% and 66.92%, respectively. In contrast, the contents of hydrocarbons decreased to 7.33% and 2.57% in 2‐year and 3‐year samples, while esters became the predominant components in 2‐year and 3‐year samples (60.55% and 87.88%, respectively). Specifically, decanoic acid.decylester and heptadecane dominated in 0‐year and 1‐year samples, respectively. Whereas, palmitic acid and ethyl ester became the most abundant volatiles in 2‐year samples, while octanoic acid and ethyl ester were the most abundant volatiles in 3‐year samples. There were eight flavor compounds in all samples, including hexadecane, heptadecane, ethyl caprylate, methyl palmitate, 3‐buten‐2‐one.4‐(2.6.6‐trimethyl‐1‐cyclohexen‐1‐yl), cyclopropyl isothiocyanate, allyl isothiocyanate, and 2,3‐dihydroxypropyl linolenic acid. According to the hierarchical cluster results (Figure 2), ZZDJ samples could be classified into two clusters, 0‐year and 1‐year samples in group 1, and 2‐year and 3‐year samples in group 2. The composition of volatile compounds in each group was somewhat more similar.

Additionally, principal component analysis (PCA) was performed to evaluate the difference in volatile profiles among ZZDJ samples. As shown in Figure 3b, PCA explained most of the volatile compounds in two components, with 68.3% and 27.1% variance by first principal component (PC1) and second principal component (PC2), respectively. Hence, PC1 and PC2 could be used to identify differences among all ZZDJ samples. It could be easily distinguished that ZZDJ samples were placed in three different regions of the score plot map, with 0‐year and 1‐year samples grouping together in the second quadrant, and 2‐year and 3‐year samples locating in the fourth and the first quadrants, respectively (Figure 3b). Then we could conclude that during the fermentation process of ZZDJ, the volatile compositions were quite similar at the starting point (0‐year) and the initial stage (1‐year), and quite different at the later stage (2‐year and 3‐year), corresponding with the hierarchical cluster results mentioned above. In the loading plot map, the further the compounds were away from the origin, the more important they were to separate samples. The closer they were to the samples’ position, the more representative they were in the corresponding samples (Huang, Fu, et al., 2019). In our study, the distribution of 0‐year and 1‐year samples in the PCA plot was relevant to heptadecane (A15), 2,6,10‐trimethylhexadecane (A16), and decanoic acid.decylester (B24). Similarly, ethyl caproate (B2), methyl palmitate (B29), and ethyl palmitate (B31) might contribute the most to the flavor of 2‐year sample, while ethyl caprylate (B8) and ethyl nonanoate (B13) might contribute the most to the flavor of 3‐year sample (Figure 3c).

### 3.2 Analysis of microbiota communities during the ZZDJ fermentation process

#### 3.2.1 Composition and change of bacterial communities

**FIGURE 4:**
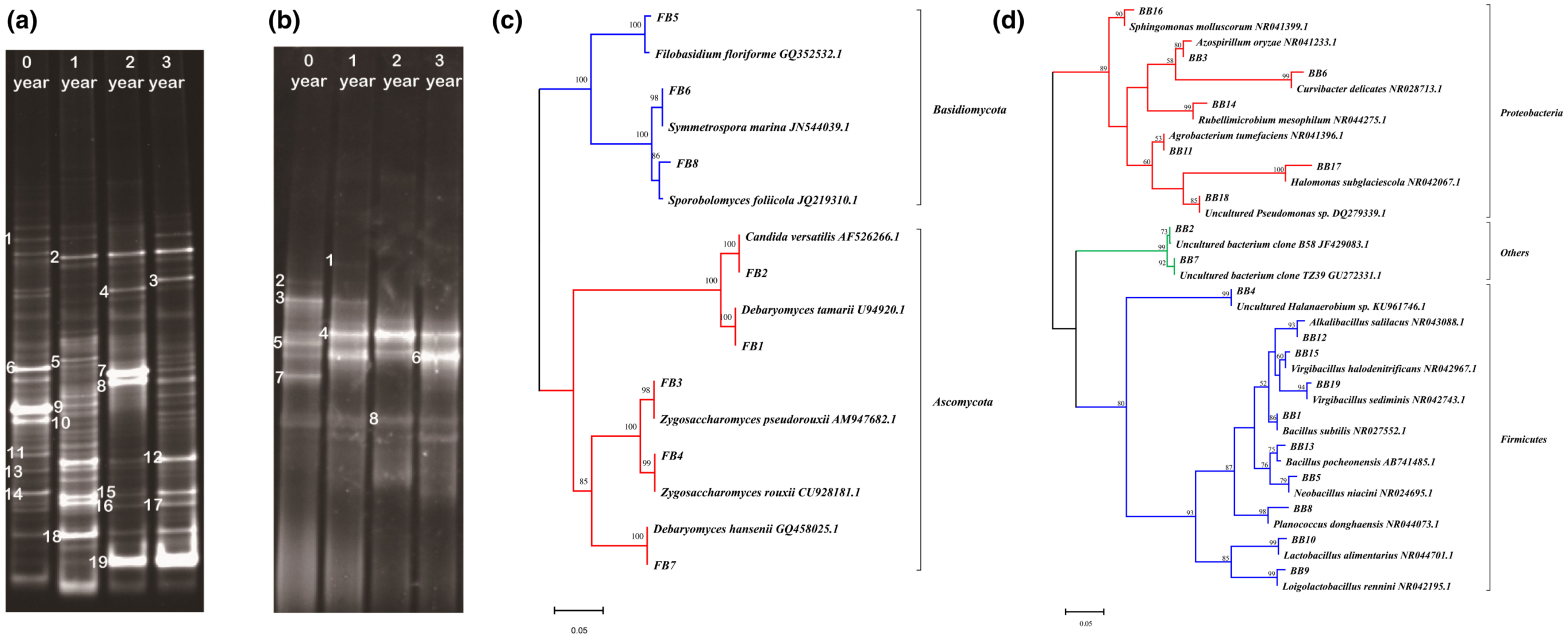
Denaturing gradient gel electrophoresis (DGGE) profiles of bacterial communities (a) and fungal communities (b) in ZZDJ samples from four different periods of fermentation. Sequencing results of numbered bands are summarized in Table 3 and Table 4. Neighbor‐joining phylogenetic trees of the bacterial species (c) and fungal species (d) in ZZDJ samples. The figures were constructed on the basis of sequencing data. Bootstrap (1000 replicates) values below 50 are not shown.

**FIGURE 5:**
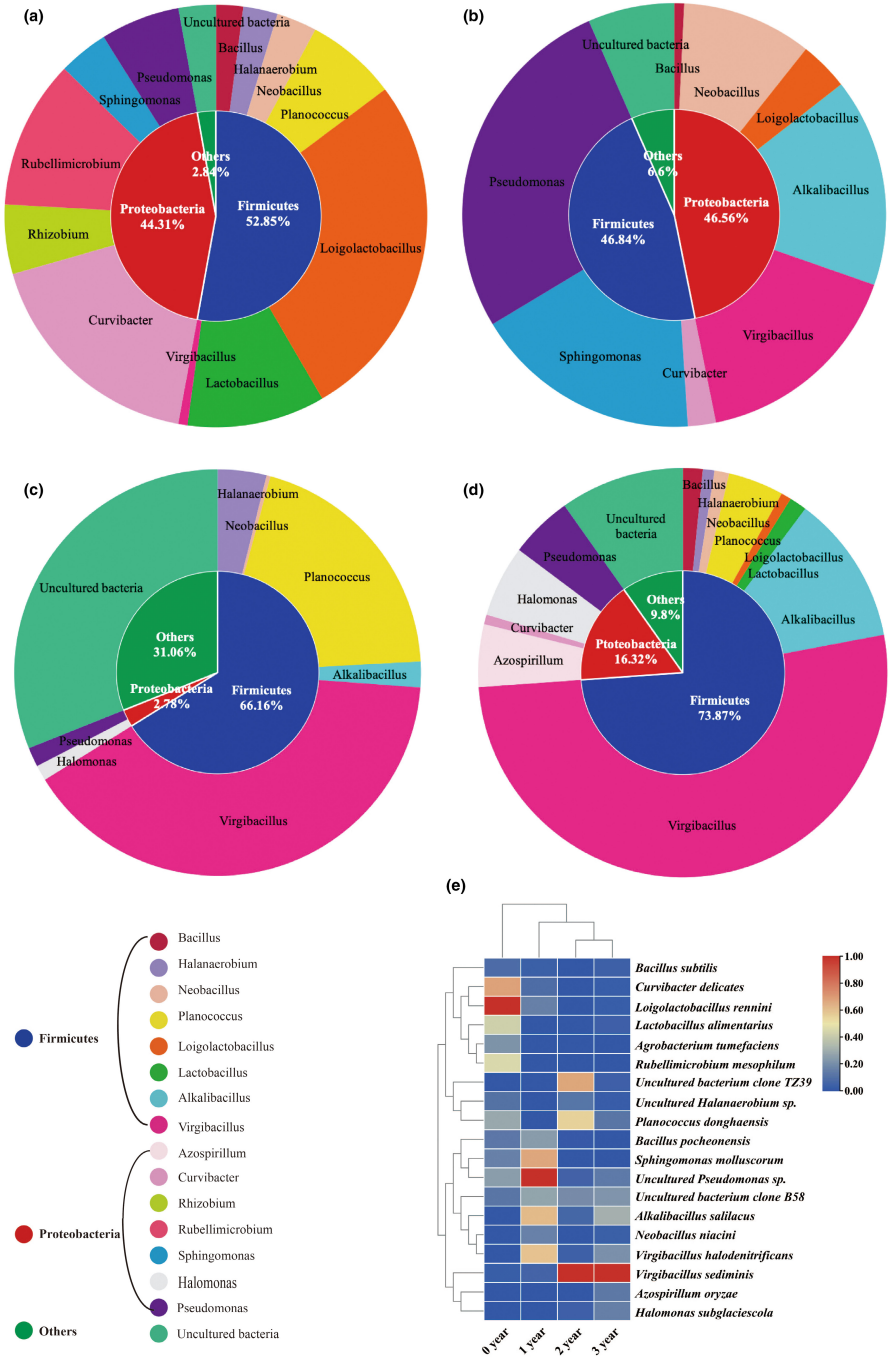
Donut plots representing the bacterial population in ZZDJ samples from (a) 0 year, (b) 1 year, (c) 2 years, and (d) 3 years. The inner and outer circles display the percentage distribution at the phylum and genus levels, respectively. Heatmap plot of the (e) bacterial species present in the four ZZDJ samples from different fermentation periods of ZZDJ. The color intensity is proportional to the relative abundance of bacterial/ fungal species.

To monitor the composition and change of bacterial communities during the ZZDJ fermentation process, PCR–DGGE analysis of bacterial 16S rDNA gene was performed. The DGGE profiles for bacterial communities of ZZDJ samples are presented in Figure 4a. The banding patterns of each sample differed in band position, number, and intensity, indicating that the constitution of bacterial communities changed during the fermentation process of ZZDJ. We calculated the diversity indices based on the relative abundances of bands (Table S2). As shown in Table 2, the Shannon–Wiener index of 1‐year sample was higher than those of other samples, indicating that the number of bacterial species reached maximum in the initial stage of the ZZDJ fermentation process. Moreover, the highest evenness index was also observed in 1‐year sample, indicating that the homogeneity of banding pattern in this stage was superior to those in other stages. In order to identify bacterial species presented during the ZZDJ fermentation process, 19 dominant bands (BB1 to BB19) were excised from the gels, sequenced and blasted in GenBank database. The results from sequence blasting and phylogenetic tree analysis are presented in Table 3 and Figure 4c. All sequences showed at least 98% similarities with their closest matched sequences in GenBank. A total of 3 phyla, 16 genera, and 19 species were identified. The bacterial population succession at different times of the ZZDJ fermentation process is shown in Figure 5. Notably, the diversity and percentage distribution of bacterial communities varied over the fermentation time at phylum and genus levels. Specifically, phylum *Firmicutes* remained dominant throughout the ZZDJ fermentation process and its relative abundance reached as high as 73.87% in 3‐year sample. Whereas, the relative abundance of phylum *Proteobacteria* decreased from 44.31% to 16.32% in 3‐year sample. At genus level, genus *Loigolactobacillus* was most abundant at the starting point of fermentation (0‐year sample), then decreased and was taken over by *Pseudomonas* in the 1‐year sample. *Virgibacillus* increased dramatically and became the most predominant genus at the later stage (the 2‐year and 3‐year samples) of fermentation. The abundance of bacterial species in each sample is illustrated in Figure 5e. The results suggested that the dominant bacteria differed as the fermentation proceeded. At the beginning of fermentation, *Curvibacter delicates*, *Loigolactobacillus rennini*, and *Lactobacillus alimentarius* were predominant, but their abundances declined to 0.77%, 0.81%, and 1.37% at the end of fermentation, respectively. Additionally, *Rubellimicrobium mesophilum* (11.4%) also dominated at the starting point of fermentation but vanished thereafter. In 1‐year sample, *Alkalibacillus salilacus*, *Virgibacillus halodenitrificans*, *Uncultured Pseudomonas* sp., and *Sphingomonas molluscorum* were abundant but were then taken over by *Uncultured bacterium clone TZ39*, *Planococcus donghaensis*, and *Virgibacillus sediminis* in 2‐year sample. At the end of fermentation, *Virgibacillus sediminis* remained dominant and *Alkalibacillus salilacus* became the major bacterium again. Furthermore, *Uncultured bacterium clone B58*, *Uncultured Pseudomonas* sp., and *Virgibacillus sediminis* were present throughout the ZZDJ fermentation process. *Rubellimicrobium mesophilum* and *Azospirillum oryzae* appeared only in 0‐year and 3‐year samples, respectively.

**TABLE 2:** Diversity indices of bacteria and fungi in different fermentation stages of Dongjian based on relative abundance of bands depicted in Figure 3.

Lane^a^	Bacterial diversity		Fungal diversity	
	Shannon–Wiener	Evenness	Shannon–Wiener	Evenness
A	2.01	0.84	1.73	0.89
B	2.20	0.86	1.27	0.79
C	1.61	0.70	0.86	0.78
D	1.97	0.73	0.83	0.75

^a^ Lanes A, B, C, and D represent samples collected from 0 year, 1 year, 2 year, and 3 year Dongjian, respectively.

**TABLE 3:** Identities of 16S rRNA sequences of bacterial DGGE bands via BLAST.

Band no.	Coding	Closest relative species^a^	GenBank accession number	Similarity rate (%)^b^
1	BB1	*Bacillus subtilis*	NR027552.1	100
2	BB 2	*Uncultured bacterium clone B58*	JF429083.1	99
3	BB 3	*Azospirillum oryzae*	NR041233.1	99
4	BB 4	*Uncultured Halanaerobium* sp.	KU961746.1	100
5	BB 5	*Neobacillus niacini*	NR024695.1	100
6	BB 6	*Curvibacter delicates*	NR028713.1	100
7	BB 7	*Uncultured bacterium clone TZ39*	GU272331.1	98
8	BB 8	*Planococcus donghaensis*	NR044073.1	98
9	BB 9	*Loigolactobacillus rennini*	NR042195.1	99
10	BB 10	*Lactobacillus alimentarius*	NR044701.1	99
11	BB 11	*Agrobacterium tumefaciens*	NR041396.1	100
12	BB 12	*Alkalibacillus salilacus*	NR043088.1	99
13	BB 13	*Bacillus pocheonensis*	AB741485.1	98
14	BB 14	*Rubellimicrobium mesophilum*	NR044275.1	99
15	BB 15	*Virgibacillus halodenitrificans*	NR042967.1	100
16	BB 16	*Sphingomonas molluscorum*	NR041399.1	99
17	BB 17	*Halomonas subglaciescola*	NR042067.1	98
18	BB 18	*Uncultured Pseudomonas* sp.	DQ279339.1	100
19	BB 19	*Virgibacillus sediminis*	NR042743.1	100

^**a**^ Only the highest homology matches are presented.

^**b**^ Identity represents % similarity shared with the sequences in the GenBank databases.

#### 3.2.2 Composition and change of fungal communities

**FIGURE 6:**
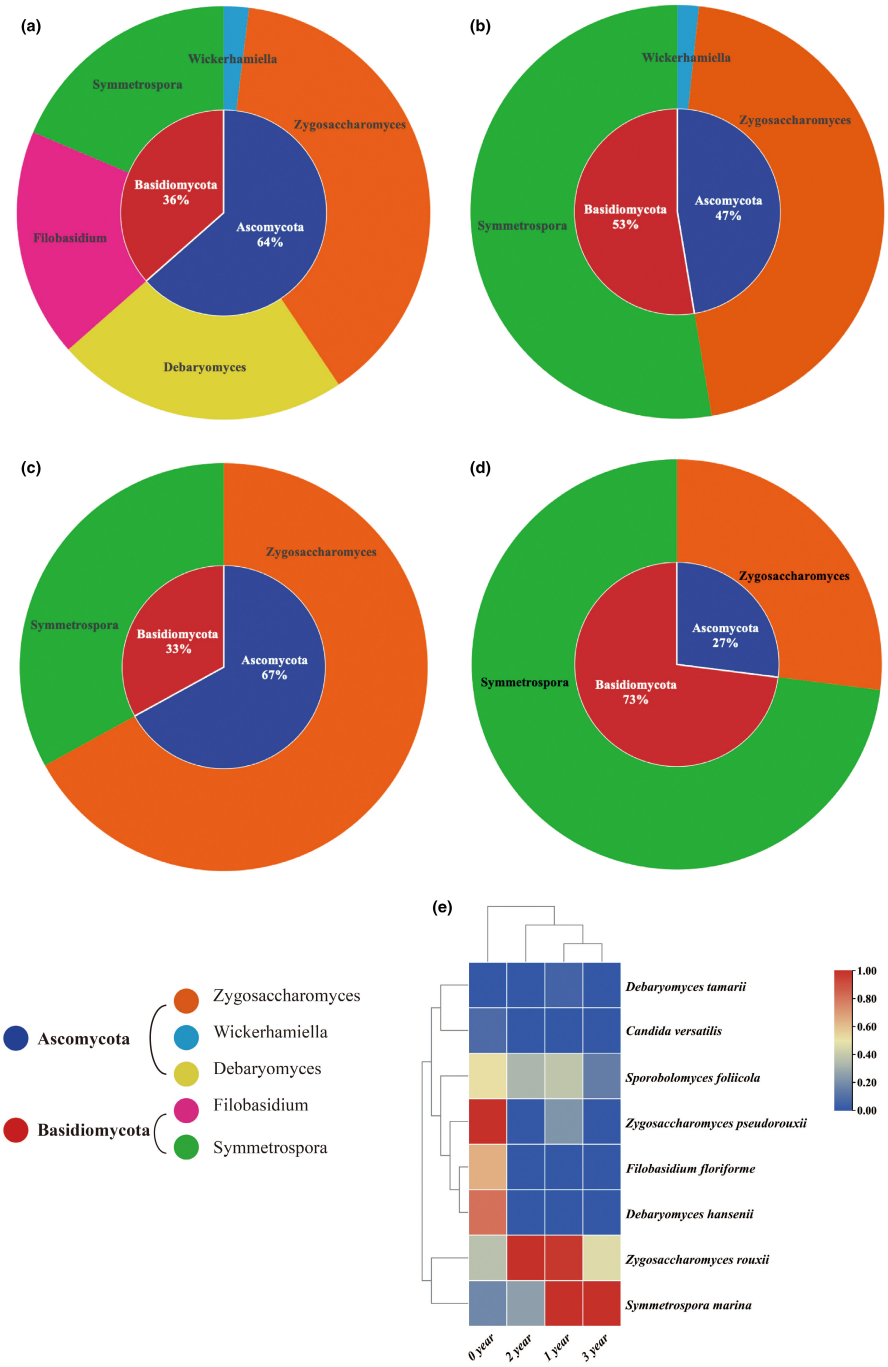
Donut plots representing the fungal population in ZZDJ samples from (a) 0 year, (b) 1 year, (c) 2 years, and (d) 3 years. The inner and outer circles display the percentage distribution at the phylum and genus levels, respectively. Heatmap plot of the (e) fungal species present in the four ZZDJ samples from different fermentation periods of ZZDJ. The color intensity is proportional to the relative abundance of bacterial/ fungal species.

The DGGE fingerprints of fungal communities during the ZZDJ fermentation process are shown in Figure 4b. Compared with bacterial DGGE profiles, the number of bands decreased dramatically, indicating that the diversity of fungi was obviously lower than that of bacteria. Notably, the banding patterns differed greatly from the 1‐year to 2‐year samples and then became stable thereafter, indicating that fungal constitution changed but then gradually became steady during the ZZDJ fermentation process. In terms of Shannon–Wiener index (Table 2), it decreased from 1.73 in 1‐year sample to 0.86 in 2‐year sample and 0.83 in 3‐year sample. The results indicated that as the fermentation time went on, the diversity of fungal communities tended to decrease, but then remained stable at the later stage (2‐year and 3‐year samples). Eight representative bands (F1–F8) of fungal DGGE profiles were excised and sequenced. The results of sequencing and evolutionary tree are represented in Table 4 and Figure 4d. All fungi belonged to two phyla: *Ascomycota* and *Basidiomycota*. Five fungal genera were found, including *Wickerhamiella*, *Zygosaccharomyces*, *Debaryomyces*, *Filobasidium*, and *Symmetrospora*. Notably, the types and quantities of fungal communities changed as the fermenting time went on (Figure 6). Specifically, *Debaryomyces* and *Filobasidium* vanished in 1‐year sample and *Wickerhamiella* disappeared in 2‐year sample. *Zygosaccharomyces* and *Symmetrospora* emerged in each sample and dominated alternatively throughout the ZZDJ fermentation process. Furthermore, the ratios of fungal species based on the relative abundances of bands (Table S2) are presented in Figure 6e. The dominant fungal species changed over the ZZDJ fermentation process. Specifically, at the starting point of fermentation, *Zygosaccharomyces pseudorouxii* was the most dominant fungus, followed by *Debaryomyces hansenii* and *Filobasidium floriforme*. *Symmetrospora marina* and *Zygosaccharomyces rouxii* become the most abundant fungi in 1‐year sanple. In the later stage of fermentation, fungal communities were consistent in type, but varied in quantity. Particularly, *Zygosaccharomyces rouxii* and *Symmetrospora marina* dominated in 2‐year sample, respectively. *Candida versatilis*, *Filobasidium floriforme*, and *Debaryomyces hanseni*i were unique to 0‐year sample, while *Debaryomyces tamarii* was special to 1‐year sample. *Zygosaccharomyces rouxii*, *Symmetrospora marina*, and *Sporobolomyces foliicola* were common fungi in all ZZDJ samples.

**TABLE 4:** Identities of 26S rRNA sequences of fungal DGGE bands via BLAST.

Band no.	Coding	Closest relative species^a^	GenBank accession number	Similarity rate (%)^b^
1	FB1	*Debaryomyces tamarii*	U94920.1	100
2	FB 2	*Candida versatilis*	AF526266.1	99
3	FB 3	*Zygosaccharomyces pseudorouxii*	AM947682.1	100
4	FB 4	*Zygosaccharomyces rouxii*	CU928181.1	100
5	FB 5	*Filobasidium floriforme*	GQ352532.1	100
6	FB 6	*Symmetrospora marina*	JN544039.1	99
7	FB 7	*Debaryomyces hansenii*	GQ458025.1	98
8	FB 8	*Sporobolomyces foliicola*	JQ219310.1	99

^a^ Only the highest homology matches are presented.

^b^ Identity represents % similarity shared with the sequences in the GenBank databases.

#### 3.2.3 Principal component analysis (PCA) based on the relative abundance of DGGE profiles

**FIGURE 7:**
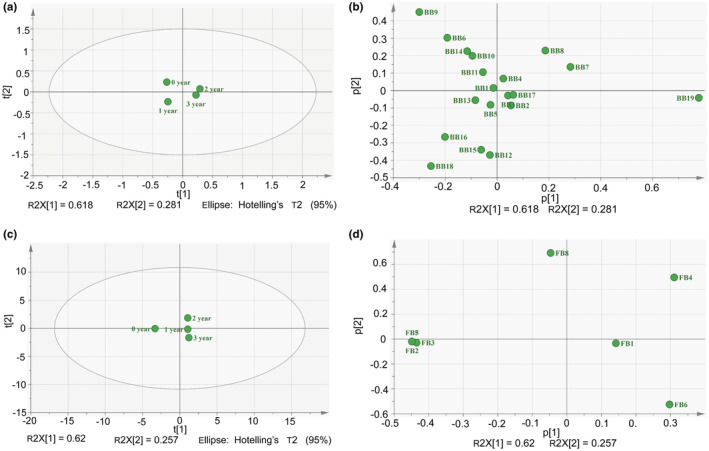
The PCA (a) score and (b) loading plots of bacteria community in 0–3‐year ZZDJ samples. PCA (c) score and (d) loading plots of fungal community in 0–3 year ZZDJ samples.

To explore the difference and similarity of microbial community structure during the ZZDJ fermentation process, PCA was performed based on the bacterial and fungal DGGE profiles. The elliptical area on the PCA plot represented 95% confidence interval (CI). As shown in Figure 7, all the samples were located within ellipse, indicating that there were no abnormal data. In terms of bacterial communities, the PCA score plots explained 89.9% of the total variance in two components (Figure 7a). Obviously, ZZDJ samples of different years were separated from each other, suggesting that bacterial community succession occurred over the fermented time. Specifically, 0‐year and 1‐year samples were located on the left quadrant, while 2‐year and 3‐year samples placed on the right quadrant. In the PCA score plot, the distances between sample points were usually used to assess the similarity of microbial communities. The distance between 2‐year and 3‐year samples was relatively close. Thus, the bacterial community structures at the later stage (2‐year and 3‐year samples) tended to be similar to some extent, but different from other years (0‐year and 1‐year samples). Meanwhile, the bacterial species accountable for the sample separation are exhibited in Figure 7b. The distribution of 0‐year sample might be due to *C. delicates* (BB6), *L. rennini* (BB9), *L. alimentarius* (BB10), and *R. mesophilum* (BB14). Likewise, the location of 1‐year sample might be relevant to *S. molluscorum* (BB16) and *Uncultured Pseudomonas* sp. (BB18). Moreover, *Uncultured bacterium clone TZ39* (BB7) and *P. donghaensis* (BB8) were strongly correlated with 2‐year sample, while *V. sediminis* (BB19) was strongly correlated with 3‐year sample.

In terms of fungal communities, PC1 and PC2 accounted for 62% and 25.7% of the total variance, and covered most of the fungal information in ZZDJ samples (Figure 7c). Notably, all the sample points were clearly separated from each other, indicating that fungal community succession also happened during the ZZDJ fermentation process. As shown in Figure 7c, 0‐year sample lay on the left quadrant, while the other samples lay on the right quadrant, indicating that the biggest change of fungal communities seemed to occur during the starting point to the first year of ZZDJ fermentation. Furthermore, 0‐year sample was mainly characterized by *Candida versatilis* (FB2), *Z. pseudorouxii* (FB3), *F. floriforme* (FB5), and *D. hansenii* (FB7). Moreover, *D. tamarii* (FB1), *Z. rouxii* (FB4), and *S. marina* (FB6) were strongly associated with 1‐year, 2‐year, and 3‐year samples, respectively (Figure 7d).

### 3.3 Statistical correlations between microbiota and volatile flavor compounds

#### 3.3.1 Correlation analysis between flavor substances and bacterial community

**FIGURE 8:**
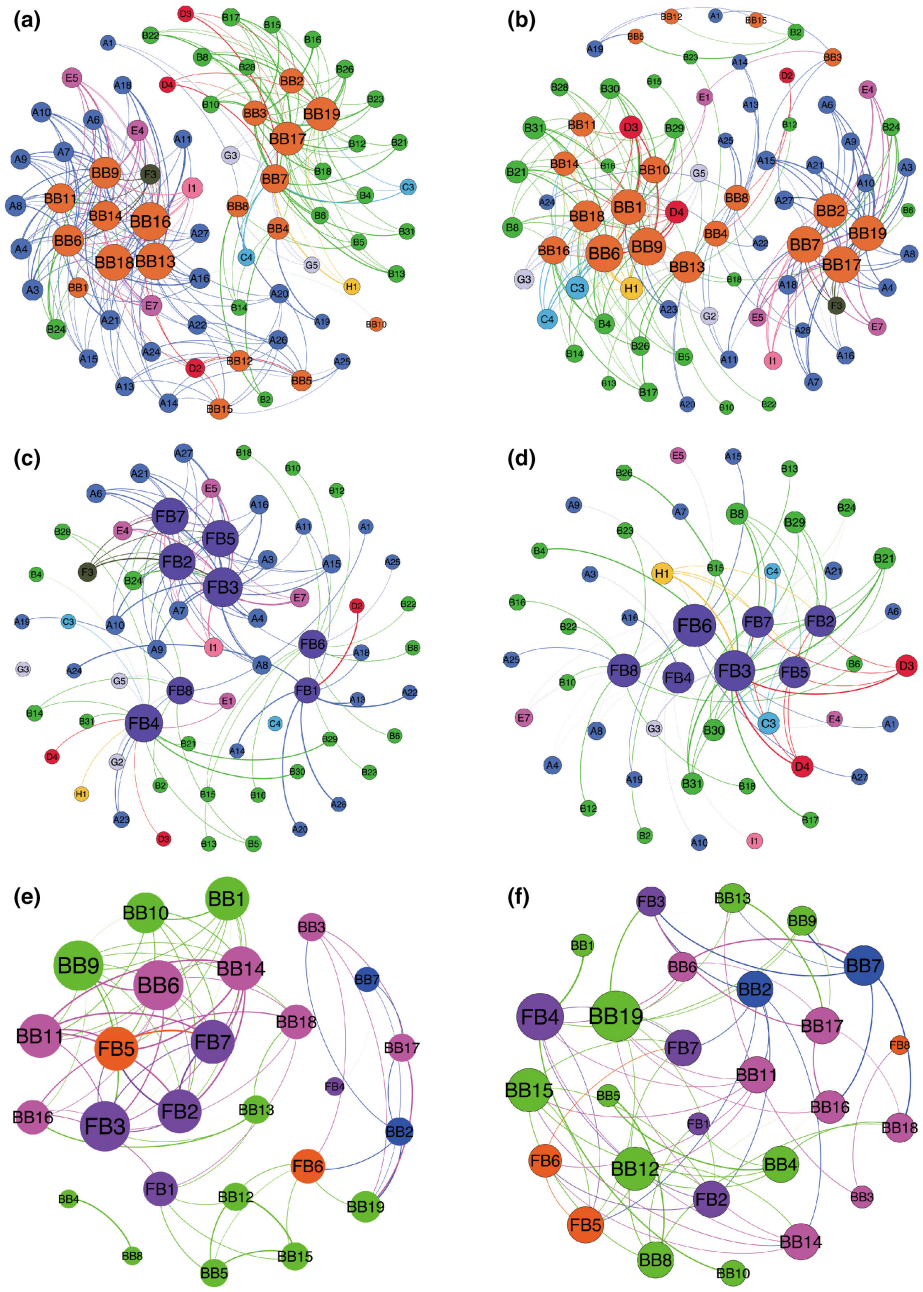
Visualization of the correlation network according to significant correlations between volatile flavor compounds and (a and b) bacteria/(c and d) fungi in ZZDJ. Network of co‐occurring microbiota in ZZDJ based on correlation analysis (e and f). Positive correlation network: a, c, e; Negative correlation network: b, d, f. Each node represents a volatile compound or a microorganism and nodes of the same color are in the same category. In addition, the size of each node is proportional to the number of connections, and line width indicates the strength of the correlation. The code representative is displayed in Table 2 and Table S2.

To explore the contribution of bacteria to flavor formation of ZZDJ, a network correlation analysis was conducted based on Spearman's rank correlations (|*ρ*| >0.6, *p* < .05; Figure 8a,b). A total of 79 nodes and 511 edges were observed. Concretely, 279 pairs of positive correlations (Figure 8a) and 232 pairs of negative correlations (Figure 8b) were confirmed. The results indicated that different bacteria contributed differently to the formation of volatile flavor compounds. Bacteria could be classified into two groups: one group positively correlated with hydrocarbons but negatively correlated with esters (except Decanoic acid.decylester, B24), while the other group was the opposite Specifically, *B. subtilis* (BB1), *C. delicates* (BB6), *L. rennini* (BB9), *Agrobacterium tumefaciens* (BB11), *Bacillus pocheonensis* (BB13), *R. mesophilum* (BB14), *S. molluscorum* (BB16), and *Uncultured Pseudomonas* sp. (BB18) had positive correlations with hydrocarbons, while *Uncultured bacterium clone B58* (BB2), *A. oryzae* (BB3), *Uncultured bacterium clone TZ39* (BB7), *Halomonas subglaciescola* (BB17), and *V. sediminis* (BB19) displayed positive relationships with esters. As the most abundant volatiles in 0‐year and 1‐year samples, Decanoic acid.decylester (B24) and heptadecane (A15) had similar relationships with bacteria, and both were positively associated with *C. delicates* (BB6), *L. rennin*i (BB9), *A. tumefaciens* (BB11), *B. pocheonensis* (BB13), *R. mesophilum* (BB14), and *S. molluscorum* (BB16). Correspondingly, the dominant substances, such as Palmitic acid, ethyl ester (B31) and octanoic acid, ethyl ester (B8) at the later fermentation stage, showed positive correlations with *Uncultured bacterium clone TZ39* (BB7), *H. subglaciescola* (BB17), and *V. sediminis* (BB19). To recognize the core flavor‐contributing bacteria during the ZZDJ fermentation process, three criteria were applied: (1) stability in all the samples; (2) the Spearman correlation coefficient |*ρ*| >0.6; and (3) the number of volatiles connected to bacteria was more than 35 **(**Wang et al., 2021 **)**. Based on these criteria, *Uncultured Pseudomonas* sp. (BB18) and *V. sediminis* (BB19) were considered as the core flavor‐producing bacteria of ZZDJ.

#### 3.3.2 Correlation analysis between flavor substances and fungal community

In the present study, network diagrams were generated to analyze the association between volatile flavor compounds and fungi (|*ρ*| >0.6, *p* < .05; Figures 8c and 7d). The network was made up of 68 nodes, which were linked by 118 positive edges and 75 negative edges. Our results revealed that the abundance of some fungi was closely associated with the contents of volatile flavor compounds. As shown in Figure 8, most hydrocarbons were in positive correlation with *C. versatilis* (FB2), *Z. pseudorouxii* (FB3), *F. floriforme* (FB5), and *D. hansenii* (FB7), but negatively correlated with *Z. rouxii* (FB4) and *S. marina* (FB6). It was worth noting that most esters showed opposite correlation. As mentioned above, Decanoic acid.decylester (B24), heptadecane (A15), Palmitic acid, ethyl ester (B31), and octanoic acid, ethyl ester (B8) were the most dominant substances in 0‐year to 3‐year samples, respectively. Decanoic acid.decylester (B24) and heptadecane (A15) both had positive correlations with *C. versatilis* (FB2), *Z. pseudorouxii* (FB3), *F. floriforme* (FB5), and *D. hansenii* (FB7). Whereas, Decanoic acid.decylester (B24) was in negative correlation with *Z. rouxii* (FB4) and *S. marina* (FB6). Heptadecane (A15) was in negative correlation with *S. marina* (FB6). Moreover, Palmitic acid, ethyl ester (B31) and octanoic acid, ethyl ester (B8) showed positive correlations with *Z. rouxii* (FB4) and *S. marina* (FB6), respectively, but were negatively correlated with *Z. pseudorouxii* (FB3), *F. floriforme* (FB5), and *D. hansenii* (FB7). The line thickness reflected the correlation strength. *Z. pseudorouxii* (FB3) had strong positive relationship with 1.4‐benzenediol.2.6‐bis(1.1‐dimethylethyl)‐ (E4), 1‐Hexadecanol,2‐methyl‐ (E5), 1‐nonadecanol (E7), and 4‐octadecylmorpholine (I1), but were negatively correlated with cyclopropyl isothiocyanate (D3), allyl isothiocyanate (D4), and 9.12.15‐octadecatrienoic acid.2.3.‐dihydroxypropylester (H1). According to the above criteria, *Z. rouxii* (FB4) and *S. marina* (FB6) might be the core flavor‐producing fungi of ZZDJ.

### 3.4 Interaction network of the ZZDJ microbial community

In order to elucidate the correlativity among microorganisms during ZZDJ fermentation, a microbial network analysis of co‐occurrence and co‐exclusion patterns was conducted based on Spearman's rank correlations (|*ρ*| >0.6, *p* < .05). Genera of two different phyla had a high co‐occurrence incidence (78.48%). Among them, the phyla *Firmicutes* and *Proteobacteria* showed the highest incidence of co‐occurrence (18.99%). The co‐occurrence incidence of genera within the same phylum was 21.52%. In positive network diagram (Figure 8e), there were three hubs (degree ≥10, where degree represents the number of one node attached to others), including *C. delicates* (BB6), *L. rennini* (BB9), and *Z. pseudorouxii* (FB3). In negative diagram (Figure 8f), it was evident that *V. sediminis* (BB19) was the hub, indicating the highest degree (degree = 10) of interaction with other microorganisms. In terms of the relationship between bacteria and fungi, *C. delicates* (BB6) and *L. rennini* (BB9) both showed positive correlations with *C. versatilis* (FB2), *Z. pseudorouxii* (FB3), *F. floriforme* (FB5), and *D. hansenii* (FB7), but negative relationships with *Z. rouxii* (FB4).

## 4 DISCUSSION

Zizhong Dongjian (ZZDJ) is a traditional pickled vegetable from Zizhong county, Sichuan province of China. It is very popular because of its attractive flavor and good taste (L. Zhang et al., 2022). ZZDJ is produced by spontaneous fermentation and the microbial community structure varies with fermentation time. Microorganisms play important roles in the quality and flavor formation of fermented food (Lee et al., 2005; Wu et al., 2015). Unfortunately, keeping stable quality and consistent flavor between different batches of ZZDJ is difficult, because like most traditionally fermented vegetables, ZZDJ is still made based on worker's experience under non‐sterile and open environments. To our knowledge, few reports have been published about the relationship between microbiota and flavor during the entire fermentation process of ZZDJ. Therefore, the dynamic changes in microbial structure and flavor compounds during ZZDJ fermentation process should be investigated to elucidate the relationship between microbiota and flavors. It is significant to reveal the most important microbes responsible for the flavor of ZZDJ. Moreover, our study might provide theoretical foundations for improving the quality and flavor of ZZDJ.

Zizhong Dongjian (ZZDJ) is manufactured by spontaneous fermentation and a number of microorganisms take part in its fermentation process. In the present study, the structures and dynamics of microbial community during the fermentation process of ZZDJ were investigated by PCR–DGGE technology. We found that the bacterial diversity reached the maximum value in the first year of fermentation. On the one hand, this might due to the fact that the first year was the beginning of transition from aerobic environment to anaerobic environment and most of the bacteria could survive at this stage. On the other hand, it might also be due to the fact that there were abundant nutrients for bacteria to grow at the initial stage of fermentation. Previous studies suggested that bacteria and fungi also participate in the fermentation processes of various vegetables (An et al., 2021; Liang, He, Wang, Song, Chen, Lin, Ji, & Li, 2020a) and the bacterial diversity is usually higher than that of fungal. An et al. (2021) analyzed the microbial diversity in 22 pickle samples and found that the number of bacterial species was significantly more than that of fungal species. Likewise, the diversity of bacteria was higher than that of fungi during the fermentation of ZZDJ, which was consistent with previous researches (An et al., 2021; Wu et al., 2015; Xiao et al., 2021). It was worth noting that all the fungal species found in ZZDJ were yeasts. Both bacterial and fungal diversities were relatively high at the starting point of ZZDJ fermentation. Raw materials, seasoning ingredients, and processing environments are the main sources of autochthonous microbiota in vegetable fermentation processes (Song et al., 2020; Zhang et al., 2022). Herein, bacterial community composition varied throughout the ZZDJ fermentation process and it changed rapidly in the second year of fermentation. Previously, Zhang et al. (2022) found that bacterial community structure changed dramatically during the third year of Dazu Dongcai fermentation. The discrepancy in these results may be closely related to several factors, such as geographical location, climatic condition, fermentation process, and raw materials (Liu, Peng, et al., 2019). Our study elucidated the relationship between microbiota and flavor formation during the fermentation process of ZZDJ. Some bacteria and fungi, such as *Uncultured Pseudomonas* sp., *V. sediminis*, *Z. rouxii*, and *S. marina*, played important roles in the flavor information of ZZDJ. These microbes could be used as starters in the production of ZZDJ by the manufacturer. However, a thorough understanding of these microbes and flavor formation of ZZDJ is needed and has to be explored in a future study.

Bacteria play an important role in the fermentation process of vegetables (Behera et al., 2020). In terms of bacterial community, *Proteobacteria* and *Firmicutes* were the dominant phyla during ZZDJ fermentation, which was in line with other fermented vegetables such as pickles, Dazu Dongcai, Nanfeng yancai, and industrial paocai (An et al., 2021; Liang et al., 2018; Liu, Li, Huang, et al., 2019; Zhang et al., 2022). In our study, 16 bacterial genera were successfully detected in ZZDJ samples and some bacterial genera, such as *Bacillus*, *Halanaerobium*, *Halomonas*, *Lactobacillus*, and *Pseudomonas*, were also found in other fermented vegetables (Liu & Tong, 2017). It has been reported that the abundance and species of bacteria change with fermentation time (Zhang, Song, et al., 2021). Herein, *Loigolactobacillus* and *Pseudomonas* were the predominant genera in 0‐year and 1‐year ZZDJ samples, respectively. Whereas, 2‐year and 3‐year ZZDJ samples were enriched in *Virgibacillus*. Zhang et al. analyze the microbial communities of 0‐year to 4‐year Dazu Dongcai samples and find that *Lactobacillus*, *Terrisporobacter*, and *Romboutsia* are the most abundant genera throughout the fermentation process. Meanwhile, Zhang et al. find that *unidentified_ Chloroplast*, *Sphingomonas*, *unidentified_Mitochondria*, and *Pseudomonas* are the predominant bacteria genera in 0‐year and 1‐year Dazu Dongcai samples (Zhang et al., 2022). Notably, not all the species of *Pseudomonas* are associated with food spoilage, and the ability of *Pseudomonas* to cause spoilage is species‐ or maybe strain‐specific (Stanborough et al., 2018). Some *Pseudomonas* species, such as *Pseudomonas fragi*, *Pseudomonas lundensis*, and *Pseudomonas fluorescens*, are commonly associated with food spoilage (Casaburi et al., 2015), whereas some *Pseudomonas* species, such as *Pseudomonas taetrolens*, are used as the producer microorganism in cheese fermentation (Alonso et al., 2015). Additionally, *Pseudomonas* are major microorganisms in several fermented foods, such as Sichuan industrial paocai and doubanjiang (Li et al., 2017; Zhao et al., 2021). Compared with bacterial community, fungal community of ZZDJ was more stable. All the fungi involved in the ZZDJ fermentation process were affiliated with *Ascomycota* and *Basidiomycota* phyla, corresponding with previous studies (An et al., 2021; Liang et al., 2018; Liu, Peng, et al., 2019; Zhang et al., 2018).

As a kind of probiotic, lactic acid bacteria (LABs) existed widely in fermented vegetables, such as kimchi, Pao cai, Suan‐cai, and Jiang‐shui (Lee et al., 2017; Liang et al., 2018; Liu, Li, Wei, et al., 2019). *Lactobacillus*, *Leuconostoc*, *Pediococcus*, *Lactococcus*, and *Weissella* are the most common LABs in fermented vegetables. Nevertheless, the predominant LABs vary in different fermented vegetables (Lee et al., 2017; Liu, Li, Wei, et al., 2019; Liu & Tong, 2017; Nguyen et al., 2013). In the present study, two kinds of LABs (*Lactobacillus* and *Loigolactobacillus*) were found in ZZDJ, which dominated at the starting point of fermentation, but their content reduced or even disappeared thereafter. From this point of view, we deduced that the growth of *Lactobacillus* and *Loigolactobacillus* was inhibited as the fermentation of ZZDJ proceeded. Unlike ZZDJ, low‐salt fermented vegetables (e.g., kimchi and Suan‐cai) have been dominated by LABs throughout the fermentation process. These differences may take place due to salt concentration. The average salt concentrations of kimchi, Suan‐cai, and ZZDJ are 3%, 2.11%, and 13%, respectively. ZZDJ has a really high salt concentration. Previous researches have shown that the salt concentration can affect bacterial composition in fermented vegetables, and high salt concentration inhibits the growth of LABs (Chang et al., 2008; Liang, He, Wang, Song, Chen, Lin, Ji, & Zhang, 2020b; Yang et al., 2019). Moreover, high salt concentration may facilitate the growth of moderate halophiles that can survive well in 3–15% sodium chloride (NaCl) environments (Kushner, 1968). Consistent with other high‐salt fermented vegetables, ZZDJ were dominated by moderate halophiles, including *Alkalibacillus*, *Virgibacillus*, *Halomonas*, and *Halanaerobium* (Liang et al., 2021; Zhang, Song, et al., 2021; Zhang, Zhang, et al., 2021). In the present study, the fungal community was more stable than the bacterial community during the fermentation process of ZZDJ. Interestingly, all the fungi involved in the fermentation process of ZZDJ were affiliated with *Ascomycota* and *Basidiomycota* phyla, corresponding with previous studies (An et al., 2021; Liang et al., 2018; Liu, Peng, et al., 2019; Zhang et al., 2018). Compared with LABs, yeasts can survive well in high salt environment because of its stronger salt tolerance (An et al., 2021).

Volatile flavor compounds are crucial components for the special taste and flavor of fermented food (Xiao et al., 2018). Nowadays, there are many reports on flavor characters in fermented food, but relatively few in ZZDJ. The unique flavor of ZZDJ makes it popular among Chinese people. Thus, we adopted the HS‐SPME‐GC–MS technique to analyze the dynamic changes of volatile compounds during the fermentation process of ZZDJ. Herein, a total of 84 volatile compounds were detected, including hydrocarbons, esters, ketones, sulfides, alcohols, aldehydes, aromatic compounds, acids, and heterocyclic compounds. In the process of ZZDJ fermentation, the number of volatile compounds reached maximum in the first year. In comparison, there are 57 volatile compounds in Nanchong Dongcai and volatile compounds reach the highest level in the third year (Yao et al., 2015). Nanchong Dongcai usually adds spices in the production process, whereas ZZDJ does not. Thus, we speculated that spices might be responsible for the difference of flavors between ZZDJ and Nanchong Dongcai. Hydrocarbons were dominant in 0‐year and 1‐year ZZDJ samples, while esters replaced hydrocarbons as the predominant compounds in 2‐year and 3‐year samples. Hydrocarbons might contribute less to the aroma of food. Nonetheless, they could act as precursors of other flavor compounds (Bontinis et al., 2012). Esters are considered as crucial contributors to the flavor of fermented vegetables, as they can impart fruity, floral, and honey aromas, even at low concentrations (Pogacic et al., 2016).

Volatile flavor compounds can be influenced by various factors, such as fermentation technology, vegetable species, regions, and microbiota. Among them, microorganisms are crucial for the formation of flavors in fermented vegetables (Liang, He, Wang, Song, Chen, Lin, Ji, & Zhang, 2020b). Therefore, more and more attention is being paid to the interaction between microbiota and flavor compounds in fermented vegetables, such as mustard tuber, potherb mustard, and radish (Liu, She, et al., 2020; Zhang, Chen, et al., 2019; Zhang, Zhang, et al., 2021). Nevertheless, there is no research about the relationship between microorganism and flavor formation during ZZDJ fermentation. In this study, *Uncultured Pseudomonas* sp. (BB18) and *V. sediminis* (BB19) were the core flavor‐producing bacteria of ZZDJ. 1‐undecen, 2‐nonaone, and 5‐methyl‐2‐hexaone were usually considered as marker compounds for the degree of spoilage and commonly attributed to the presence of spoilage‐associated *Pseudomonas* (Stanborough et al., 2018). These substances were not detected in our study, thus we speculated that *Uncultured Pseudomonas* sp. (BB18) did not have spoilage potential and might not categorize as spoilage‐associated *Pseudomonas*. Conversely, it may contribute to flavor formation of ZZDJ. *Pseudomonas* species are commonly detected in daily fermented vegetables and can facilitate the production of hydrocarbons, ketones, esters, sulfides, and aldehydes (Guo et al., 2021; Lee et al., 2017; Liu et al., 2018). The 0‐year and 1‐year ZZDJ samples dominated by *Pseudomonas* were rich in hydrocarbons. This result was in agreement with those of previous studies, which showed that *Pseudomonas* has the ability to produce higher levels of hydrocarbons (Zhang, Wei, et al., 2019). Zhu et al. (2022) explore microbial community succession in Jiang‐flavored Daqu and find that *Virgibacillus* is one of the signature microbes in the fermented Daqu products. Similarly, we found that *Virgibacillus* was the predominant genus at the later stage (the second and the third years) of ZZDJ fermentation. Recently, more and more *Virgibacillus* species are isolated. For example, *Virgibacillus kimchi* sp., *Virgibacillus alimentarius* sp., and *Virgibacillus kapii* are isolated from kimchi, salt‐fermented seafood, and Thai shrimp paste, respectively (Daroonpunt et al., 2016; Kim et al., 2011; Oh et al., 2017). However, there are few studies that analyzed the correlation between *Virgibacillus* species and flavor compounds. As a kind of moderately halophilic bacterium, *V. sediminis* is first isolated from a salt lake in China (Chen et al., 2009). However, the existence of *V. sediminis* in fermented vegetables has not been reported yet. Herein, *V. sediminis*, which dominated at the later stage of ZZDJ fermentation, displayed positive relationship with most esters. As a potential limitation of our study, we did not isolate *V. sediminis* from ZZDJ nor provide a comprehensive analysis of correlation between *V. sediminis* and flavor compounds. This may be done in our future research. Fungi, especially yeasts, also take part in flavor development of fermented food. Previous reports indicate that *Z. rouxii* can promote flavor formation of fermentation food (Devanthi et al., 2018; Lee et al., 2013; Liu, Wang, et al., 2020). Due to its high salt tolerance, *Z. rouxii* is widely applied in the production of fermented food. In the present study, *Z. rouxii* was consistently predominant throughout the ZZDJ fermentation process. PCR–DGGE has been widely used to investigate microbial diversity and dynamics of fermented food both quickly and economically. Compared with PCR–DGGE, next‐generation sequencing (NGS) technology could provide a deeper and more precise evaluation of complex microbiota. Potential limitations of our study included that we used only one column for flavor analysis and did not use NGS analysis in our study, and this may be conducted in our future studies.

## 5 CONCLUSION

In the present study, the microbial community succession, the volatile compound dynamics, and their correlation during the fermentation process of ZZDJ were explored. We found that *Loigolactobacillus*, *Pseudomonas*, and *Virgibacillus* were the most abundant bacteria in different phases of ZZDJ fermentation. Meanwhile, *Zygosaccharomyces* and *Symmetrospora* dominated alternatively throughout the ZZDJ fermentation process. Furthermore, a total of 84 volatile compounds, including 27 hydrocarbons, 33 esters, 5 ketones, 4 sulfides, 6 alcohols, 2 aldehydes, 5 aromatic compounds, 1 acid, and 1 heterocyclic compound, were detected. A correlation model was established to reveal the potential relationship between microbiota and volatile flavor compounds. Generally, these findings are meaningful because they contribute to the understanding of microbiota and volatile compounds in fermented ZZDJ and reveal the most important microorganisms responsible for ZZDJ flavor. Moreover, these results may be helpful to improve the production technique of ZZDJ.

## AUTHOR CONTRIBUTIONS

**Zhang Li:** Methodology, visualization, investigation, data curation, writing‐original draft. **Miao Wang:** Methodology, visualization, investigation, formal analysis, data analysis, writing‐original draft. **Zhirong Yang:** Resources, supervision, project administration, writing‐review & editing. All authors have read and agreed to the published version of the manuscript.

## FUNDING INFORMATION

This research did not receive any specific grant from funding agencies in the public, commercial, or not‐for‐profit sectors.

## CONFLICT OF INTEREST STATEMENT

The authors declare that they have no conflict of interests.

## Supporting information


Table S1.

Table S2.

## Data Availability

The data that support the findings of this study are available from the corresponding author upon reasonable request.
