# Supplementary material for: Analysis of the association between microbiota and flavor formation during Zizhong Dongjian fermentation process
Source: Food Sci Nutr. 2024 Oct 17;12(11):9493–510. doi: 10.1002/fsn3.4460 (PMC11606816; doi:10.1002/fsn3.4460)
Supplement: Supplementary file 1 — Table S1. [file FSN3-12-9493-s002.docx]

**Table S1** Volatile flavor compounds detected in Sichuan Dongjian samples from different periods.

| Families compounds | Code | Compounds | Retention Time(min) | Relative content (%) | | | |
| --- | --- | --- | --- | --- | --- | --- | --- |
|  |  |  |  | 0 year | 1year | 2year | 3 year |
| Hydrocarbons | A1 | 3-carene | 10.87 | 2.15 | 1.86 | 2.69 | ND |
|  | A2 | Tetradecane | 25.02 | ND | ND | ND | 0.33 |
|  | A3 | caryophyllene | 25.63 | *5.43* | 2.68 | ND | ND |
|  | A4 | Thujopsene | 25.92 | 3.21 | 0.35 | ND | ND |
|  | A5 | Tetradecane.2.6.10-trimethyl- | 26.58 | 0.08 | ND | 0.35 | 0.34 |
|  | A6 | heptadecane.2.6.10.14-tetramethyl- | 26.6 | 2.01 | 0.63 | ND | ND |
|  | A7 | (+)-epi-bicyclosesquiphenllandrene | 26.72 | 3.92 | 2.71 | ND | ND |
|  | A8 | Pentadecane | 27.53 | 4.79 | 3.86 | ND | ND |
|  | A9 | Hexadecane.1.1-bis(dodecyloxy)- | 28.76 | 7.91 | 0.44 | ND | ND |
|  | A10 | Pentadecane.2-methyl- | 29.04 | 2.17 | 0.72 | ND | ND |
|  | A11 | hexadecane | 29.9 | *4.11* | 8.29 | 0.32 | 0.46 |
|  | A12 | Cedrane.8-propoxy- | 30.05 | 0.03 | ND | 0.26 | ND |
|  | A13 | 2,6,10-trimethylpentadecane | 31.01 | ND | 3.83 | ND | ND |
|  | A14 | Hexadecane,4-methyl- | 31.32 | ND | 1.23 | ND | ND |
|  | A15 | heptadecane | 32.14 | 12.01 | 10.09 | 0.82 | 0.47 |
|  | A16 | 2,6,10-trimethylhexadecane | 32.27 | 10.81 | 8.23 | ND | 0.31 |
|  | A17 | 17-Pentatriacontene | 32.42 | ND | 0.92 | ND | ND |
|  | A18 | Heptadecane.9-hexyl- | 33.49 | 0.14 | 1.03 | ND | ND |
|  | A19 | Tetratetracontane | 34.24 | *0.71* | 0 | 0.85 | ND |
|  | A20 | Octadecane | 34.27 | ND | 5.81 | ND | ND |
|  | A21 | phytan | 34.47 | 7.14 | 5.78 | ND | ND |
|  | A22 | eicosane | 38.24 | ND | 2.87 | ND | ND |
|  | A23 | heneicosane | 40.1 | ND | 0.79 | 1.73 | ND |
|  | A24 | Docosane | 41.95 | ND | 1.54 | ND | ND |
|  | A25 | cubebene | 26.29 | 0.31 | 0.66 | ND | 0.66 |
|  | A26 | Azulene.1.2.3.4.5.6.7.8-octahydro-1.4-dimethyl-7-(1-methylethylidene)- | 28.18 | ND | 1.1 | ND | ND |
|  | A27 | 6-aminoundecane | 31.58 | *3.01* | 1.5 | 0.31 | ND |
| Esters | B1 | Pentanoic acid.ethylester | 9.83 | ND | ND | ND | 0.84 |
|  | B2 | ethyl caproate | 13.29 | 0.09 | 0 | 8.73 | ND |
|  | B3 | Heptanoic acid.methylester | 14.12 | ND | ND | 0.25 | ND |
|  | B4 | heptanoic acid, ethyl ester | 16.5 | ND | ND | 6.27 | 1.03 |
|  | B5 | octanoic acid, methyl ester | 17.32 | ND | ND | 1.41 | ND |
|  | B6 | Hexanoic acid.2-methylpropylester | 18.12 | ND | ND | ND | 1.27 |
|  | B7 | 7-Octenoic acid.ethylester | 19.24 | ND | ND | ND | 0.57 |
|  | B8 | Ethyl caprylate | 19.51 | *0.12* | 0.53 | 2.21 | 27.85 |
|  | B9 | nonanoic acid, methyl ester | 20.28 | ND | ND | ND | 0.64 |
|  | B10 | Isopentyl hexanoate | 21 | ND | ND | ND | 1.9 |
|  | B11 | Hexanoic acid.2-methylbutylester | 21.08 | 0.03 | ND | ND | 0.4 |
|  | B12 | 8-Nonenoic acid.ethylester | 22.07 | ND | ND | ND | 1.73 |
|  | B13 | ethyl nonanoate | 22.3 | ND | ND | ND | *14.17* |
|  | B14 | decanoic acid, methyl ester | 23.04 | ND | ND | 2.21 | 0 |
|  | B15 | Pentyloctanoate | 23.67 | ND | ND | ND | 2.39 |
|  | B16 | Ethyltrans-4-decenoate | 24.72 | ND | ND | ND | 1.41 |
|  | B17 | decanoic acid, ethyl ester | 24.92 | ND | ND | 0.95 | 2.91 |
|  | B18 | Octanoic acid.3-methylbutylester | 26.21 | ND | ND | ND | 3.56 |
|  | B19 | Nerolidyl acetate | 26.49 | 0.19 | 0.31 | ND | 0 |
|  | B20 | Undecanoic acid.ethylester | 27.41 | ND | ND | ND | 0.94 |
|  | B21 | 2（4H)-benzofuranone,5,6,7,7a-tetrahydro-4,4,7a-trimethyl- | 28.34 | ND | 0.45 | 1.04 | 0.6 |
|  | B22 | Nonanoic acid.pentylester | 28.62 | ND | ND | ND | 1.3 |
|  | B23 | Dodecanoic acid.ethylester | 29.77 | ND | ND | ND | 1.26 |
|  | B24 | Decanoic acid, decylester | 30.78 | *12.62* | 9.46 | ND | ND |
|  | B25 | Tridecanoic acid.12-methyl-methylester | 32.67 | ND | ND | 0.26 | ND |
|  | B26 | Tetradecanoic acid.ethylester | 34.14 | ND | ND | 1.17 | 2.74 |
|  | B27 | Phthalic acid.butyltetradecylester | 35.72 | ND | 0.76 | ND | ND |
|  | B28 | 7.10.13-Hexadecatrienoic acid.methylester | 36.28 | ND | 0 | 1.43 | ND |
|  | B29 | Methyl palmitate | 36.83 | 0.71 | 1.67 | 9.18 | 1.49 |
|  | B30 | 6.9.12.15-Docosatetraenoic acid.methylester | 37.63 | ND | 0.59 | 3.85 | 0.43 |
|  | B31 | Ethyl palmitate | 38.15 | ND | 1.95 | 20.67 | 18.45 |
|  | B32 | 9.12.15-Octadecatrienoic acid.ethylester.(ZZZ) | 41.22 | ND | ND | 0.6 | ND |
|  | B33 | 7.10-Octadecadienoic acid.methylester | 40 | ND | ND | 0.32 | ND |
| Ketones | C1 | Cyclohexanone,2,2,6-trimethyl- | 14.38 | ND | ND | ND | 0.47 |
|  | C2 | trans-geranylacetone | 26.37 | ND | ND | 0.46 | ND |
|  | C3 | 3-Buten-2-one.4-(2.6.6-trimethyl-1-cyclohexen-1-yl) | 27.26 | 0.21 | 1.72 | 4.86 | 2.81 |
|  | C4 | 2-Pentadecanone.6.10.14-trimethyl- | 35.19 | ND | ND | 0.93 | 0.71 |
|  | C5 | 7.9-Di-tert-butyl-1-oxaspiro(4.5)deca-6.9-diene-2.8-dione | 36.73 | ND | ND | 0.94 | ND |
| Sulfides | D1 | dimethyl trisulfide | 12.05 | ND | 0.32 | 0.29 | ND |
|  | D2 | tert-Hexadecanethiol | 33.09 | ND | 1.57 | ND | ND |
|  | D3 | cyclopropyl isothiocyanate | 20.19 | 0.03 | 0.45 | 0.95 | 0.6 |
|  | D4 | allyl isothiocyanate | 20.73 | ND | 0.75 | 6.15 | 1.31 |
| Alcohols | E1 | alcohol | 1.94 | 0.079 | 0.57 | 2.53 | ND |
|  | E2 | 2,6-dimethylcyclohexanol | 16.76 | ND | ND | 0.24 | ND |
|  | E4 | 1.4-Benzenediol.2.6-bis(1.1-dimethylethyl)- | 31.99 | 2.01 | 1.06 | ND | ND |
|  | E5 | 1-Hexadecanol.2-methyl- | 33.39 | 1.47 | 1.09 | ND | ND |
|  | E6 | Phytol | 38.41 | ND | ND | 0.77 | ND |
|  | E7 | 1-Nonadecanol | 31.15 | *3.47* | *0.98* | ND | ND |
| Aldehydes | F1 | benzeneacetaldehyde | 14.68 | ND | ND | ND | *0.34* |
|  | F3 | Dodecanal | 25.27 | *1.15* | *0.47* | ND | ND |
| Aromatic compounds | G2 | Phenol.2.4-bis(1.1-dimethylethyl)- | 27.85 | ND | 0.59 | 3.42 | ND |
|  | G3 | Butylated Hydroxytoluene | 27.88 | ND | ND | 2.71 | 0.77 |
|  | G4 | 9.10-Ethanoanthracene.9.10-dihydro-11.12-diacetyl- | 33.82 | ND | ND | 0.33 | 0 |
|  | G5 | 1-hydroxy-2-acetyl-4-methylbenzene | 22.76 | ND | ND | 1.24 | ND |
|  | G6 | 1.1-Biphenyl.3.4-diethyl- | 31.76 | 0.13 | 0.6 | ND | ND |
| Acids | H1 | 2,3-dihydroxypropyl linolenic acid | 41.37 | *0.23* | 0.37 | 5.63 | 0.47 |
| Heterocyclic compounds | I1 | 4-octadecylmorpholine | 39.95 | 6.31 | 5.76 | ND | ND |

ND: non detected.
