# Supplementary material for: Analysis of the association between microbiota and flavor formation during Zizhong Dongjian fermentation process
Source: Food Sci Nutr. 2024 Oct 17;12(11):9493–510. doi: 10.1002/fsn3.4460 (PMC11606816; doi:10.1002/fsn3.4460)
Supplement: Supplementary file 2 — Table S2. [file FSN3-12-9493-s001.docx]

**Table S2** Relative abundance of various bacteria and fungi in Dongjian.

| **Band** | **Relative abundance** | | | |
| --- | --- | --- | --- | --- |
|  | **0 year** | **1 year** | **2 year** | **3 year** |
| **Bacteria** |  |  |  |  |
| BB1 | 0.020830 | 0.007588 | 0 | 0.015718 |
| BB2 | 0.028414 | 0.065988 | 0.065464 | 0.084295 |
| BB3 | 0 | 0 | 0 | 0.049410 |
| BB4 | 0.026336 | 0 | 0.038827 | 0.009119 |
| BB5 | 0 | 0.038815 | 0 | 0.011594 |
| BB6 | 0.177195 | 0.021339 | 0 | 0.007696 |
| BB7 | 0 | 0 | 0.245168 | 0.013748 |
| BB8 | 0.069721 | 0 | 0.199421 | 0.043893 |
| BB9 | 0.268354 | 0.037547 | 0 | 0.008120 |
| BB10 | 0.105491 | 0 | 0 | 0.013663 |
| BB11 | 0.053075 | 0 | 0 | 0 |
| BB12 | 0 | 0.159129 | 0.020394 | 0.118169 |
| BB13 | 0.030822 | 0.060417 | 0.003061 | 0 |
| BB14 | 0.113986 | 0 | 0 | 0 |
| BB15 | 0 | 0.152765 | 0.013762 | 0.078810 |
| BB16 | 0.038180 | 0.174087 | 0 | 0 |
| BB17 | 0 | 0 | 0.012371 | 0.056579 |
| BB18 | 0.060641 | 0.270195 | 0.015399 | 0.049562 |
| BB19 | 0.006953 | 0.012129 | 0.386132 | 0.439625 |
| **Fungi** |  |  |  |  |
| FB1 | 0 | 0.016682 | 0 | 0 |
| FB2 | 0.019783 | 0 | 0 | 0 |
| FB3 | 0.288399 | 0.075224 | 0 | 0 |
| FB4 | 0.097759 | 0.381626 | 0.673272 | 0.266962 |
| FB5 | 0.178752 | 0 | 0 | 0 |
| FB6 | 0.046950 | 0.391103 | 0.141764 | 0.656012 |
| FB7 | 0.229168 | 0 | 0 | 0 |
| FB8 | 0.139189 | 0.135364 | 0.184964 | 0.077026 |

Bands are numbered according to Figure 3.
